# Expression of Concern: Chemopreventive Activity of *Ferulago angulate* against Breast Tumor in Rats and the Apoptotic Effect of Polycerasoidin in MCF7 Cells: A Bioassay-Guided Approach

**DOI:** 10.1371/journal.pone.0347381

**Published:** 2026-04-16

**Authors:** The PLOS One Editors

After this article [1] was published, concerns were raised regarding results presented in Figs 6, 9, and 12.

Specifically:

- The Fig 6A Caspase 3 panel appears to partially overlap with the Fig 6B Caspase 3 panel despite being used to represent different experimental conditions.
- The following panels appear similar despite being used to represent different experimental results:
  - Fig 9A in [1] and Fig 10 Control in [2,3]
  - Fig 12 p21 and Fig 12 p27 in [1]
- Fig 12 Caspase 7 lanes 1 and 2 appear more similar than would be expected from independent results

In addition to the above concerns, the tumor volume for group II in Table 2 is reported as 203181 mm^3^ which exceeds both internationally accepted animal welfare standards and reasonably expected tumor volumes in rats.

PLOS is investigating the concerns in accordance with COPE guidance and journal policies, but we anticipate a delay based on the initial response from a corresponding author. Meanwhile, the *PLOS One* Editors issue this Expression of Concern.
